# Supplementary material for: Molecular basis of quorum-sensing signal transduction by CqsS and its inhibition by CqsA, the autoinducer synthase
Source: Proc Natl Acad Sci U S A. 2026 Nov 2;123(45):e2617274123. doi: 10.1073/pnas.2617274123 (PMC13729885; doi:10.1073/pnas.2617274123)
Supplement: Supplementary file 1 [file pnas.2617274123.sapp.pdf]

# Supporting Information for

## Molecular basis of quorum-sensing signal transduction by CqsS and its inhibition by CqsA the autoinducer synthase

Mengchan Liu[1, *], Yufan Yang[1, *], Julie S. Valastyan[2,3 *], Yuru Zhang[1], Grace A. Beggs[2], Yanhong Liu[1], Zhenhua Wang[1], Bonnie L. Bassler[2, 3, #], Hongwu Qian[1,#]

[1]Department of Cardiology, The First Affiliated Hospital of USTC, MOE Key Laboratory for Membraneless Organelles and Cellular Dynamics, Hefei National Research Center for Interdisciplinary Sciences at the Microscale, Division of Life Sciences and Medicine, University of Science and Technology of China, Hefei 230027, China

[2]Department of Molecular Biology, Princeton University, Princeton, New Jersey, United States of America

[3]Howard Hughes Medical Institute, Chevy Chase, Maryland, United States of America

[*]These authors contributed equally to this work.

[#]To whom correspondence should be addressed: bbassler@princeton.edu and hongwuqian@ustc.edu.cn.

**This PDF file includes:**

Supplementary Materials and Methods
Figures S1 to S13
Table S1
Legend for Movies S1 to S2

**Other supporting materials for this manuscript include the following:**

Movies S1 to S2

## Supplementary Materials and Methods

### Bacterial strains and growth conditions

*E. coli* DH5α competent cells (Alpalifebio) were used for plasmid subcloning. *E. coli* BL21 (DE3) cells (Alpalifebio) were used for the expression and purification of CqsS, CqsA, and LuxU. *E. coli* were grown in Luria-Bertani (LB) medium (1% tryptone, 1% NaCl, and 0.5% yeast extract). Unless otherwise specified, all liquid cultures were aerobically grown at 37°C. When necessary, kanamycin (50 μg/mL), ampicillin (100 μg/mL), and/or streptomycin (40 μg/mL) was added to the broth.

### Cloning of *cqsS*, *cqsA*, and *luxU*

The *V. harveyi cqsS* gene was cloned from *V. harveyi* N8T11 and inserted into the pET22b vector with C-terminal Strep and FLAG tags fused in tandem for cryo-EM sample preparation, autophosphorylation, and phospho-relay assays. To validate the *cis* phosphorylation of CqsS H1 residues and *trans* phospho-transfer from CqsS H1 to D1 residues, the *mbp* gene and 10×His tag were fused in tandem to the C-terminus of the *cqsS* gene and inserted into the pRSFDuet vector. The *cqsA* gene was also cloned with an N-terminal FLAG fusion into the pRSFDuet vector for cryo-EM sample preparation and with an N-terminal 10×His fusion into the same vector for pull-down assays. The *luxU* gene was cloned from *V. harveyi* N8T11 and inserted into the pET22b vector with an N-terminal 10×His fusion. All mutants in *V. harveyi* genes were generated via a standard two-step PCR-based strategy.

**Protein expression and purification**

*E. coli* BL21 (DE3) transformed with the desired plasmid was cultured in LB medium supplemented with antibiotics at 37°C, and 1 mM isopropyl β-D-thiogalactoside (IPTG) was added when the $OD_{600}$ reached 0.8. After further culture at 37°C for 4 h, cells were collected and resuspended in lysis buffer (25 mM Tris pH 8.0, 150 mM NaCl) supplemented with 1 mM phenylmethanesulfonylfluoride (PMSF).

[Purification of CqsS proteins]

Following sonication, cell debris was removed via centrifugation at 8,000 × g at 4°C for 10 min. The clarified supernatant was subjected to ultracentrifugation at 100,000 × g at 4°C for 1 h. Subsequently, the membrane pellet was resuspended in lysis buffer containing protease inhibitor cocktail (Amresco). For purification, suspensions were solubilized with 1% (w/v) lauryl maltose neopentyl glycol (LMNG, Anatrace) at 4°C for 2 h, followed by centrifugation at 20,000 × g for 1 h. Supernatants were collected and applied to Strep-Tactin® Sepharose® (IBA), followed by rinse with buffer A containing 25 mM Tris (pH 8.0), 150 mM NaCl, and 0.005% (w/v) LMNG and elution with buffer A plus 2.5 mM desthiobiotin (Macklin). Resulting eluates were loaded onto anti-FLAG M2 resin (Sigma). The resin was rinsed with buffer A, and the target protein was eluted with buffer A plus 0.2 mg/mL FLAG peptide. Eluates were concentrated and further purified by size exclusion chromatography (SEC, Superose™ 6 Increase 10/300 GL, Cytiva) in buffer A. Peak fractions were collected and concentrated to approximately 11 mg/mL for cryo-sample preparation. All CqsS variants used here

were purified exclusively with Strep-Tactin® Sepharose® following the identical protocol described above.

[Purification of CqsS chimeras]

The CqsS-Strep and CqsS-MBP chimeras were purified via tandem affinity purification via the Strep tag on CqsS-Strep and the 10×His tag at the C-terminus of CqsS-MBP. Following sonication, the cell membrane was prepared as above. The resulting membrane proteins were extracted with 1% LMNG at 4°C for 2 h, followed by centrifugation at 20,000 × g for 1 h. Supernatants were collected and applied to Strep-Tactin® Sepharose®, followed by rinse with buffer A and elution with buffer A plus 2.5 mM desthiobiotin. Eluates were loaded onto Ni-NTA agarose (Qiagen). After rinsing with buffer A, the chimeric proteins were eluted with buffer A plus 250 mM imidazole. Eluates were concentrated and further purified by SEC in buffer A. The peak fractions were collected for phosphorylation assays.

[Purification of CqsS-CqsA-CAI-1 complexes]

Following sonication, the cell membrane was prepared via a protocol similar to that used for CqsS purification. The complexes were extracted with 1% LMNG at 4°C for 2 h, followed by centrifugation at 20,000 × g for 1 h. Supernatants were collected and applied to Strep-Tactin® Sepharose®. After rinsing with buffer A, complexes were eluted with buffer A plus 2.5 mM desthiobiotin. Eluates were subjected to SEC (Superose™ 6 Increase 10/300 GL, Cytiva) in buffer A. The peak fractions were

collected and concentrated for cryosample preparation, which was used for structural determination of CqsS at the CqsA-bound (CqsS-CqsA) and CAI-1-bound (CqsS-CAI-1) states. To obtain the CqsS-CAI complex in the absence of CqsA, the F298A/K302A mutations were introduced into CqsA to disrupt the CqsS-CqsA interaction while maintaining CAI-1 production. This CqsA variant was co-expressed with WT CqsS, and CqsS bound to CAI-1 was subsequently purified using a protocol identical to that described above. This resulting sample was used to determine the structure of CqsS-CAI-1 complex, which is designated CqsS-CAI-1 (no CqsA).

[Purification of CqsA proteins]

Following sonication, the cell debris was pelleted via centrifugation at 20,000 × g at 4°C for 1 h. The clarified supernatant was collected and applied to Ni-NTA agarose. After rinsing with lysis buffer plus 25 mM imidazole, the proteins were eluted with lysis buffer plus 250 mM imidazole. Eluates were subjected to ion exchange chromatography (Source™ 15Q, Cytiva) for further purification.

[Purification of LuxU protein]

Following sonication, cell debris was pelleted by centrifugation at 20,000 × g at 4°C for 1 h. The supernatant was collected and applied to Ni-NTA agarose resin. After rinsing with buffer B (50 mM Tris pH 8.0, 200 mM NaCl, 5 mM *β*-mercaptoethanol, and 5 mM $MgCl_2$) plus 20 mM imidazole, LuxU was eluted with buffer B plus 250 mM imidazole. The eluate was subjected to ion exchange chromatography and SEC

(Superdex™ 200 Increase 10/30 GL, Cytiva) for further purification.

### Cryo-EM sample preparation

Cryo-EM grids were prepared via Thermo Fisher Vitrobot Mark IV. Quantifoil R1.2/1.3 Cu grids were glow-discharged with air in a PDC-32G-2 plasma cleaner (Harrick) with mid-force for 85 s. Aliquots of 3.5 μL of purified CqsS or CqsS-CqsA complex, concentrated to approximately 11 mg/mL, were applied to the glow-discharged grids. After blotting with filter paper for 3.5 s (100% humidity and 8°C), the grids were plunged into liquid ethane cooled with liquid nitrogen. To capture the ATPγS-bound state, CqsS was combined with 5 mM ATPγS and 10 mM $MgCl_2$ on ice for 1 h before grid preparation.

Grids were loaded into a Titan Krios (FEI) electron microscope operating at 300 kV equipped with a BioQuantum energy filter and a K3 direct electron detector (Gatan). Images were automatically collected with EPU in super-resolution mode. Defocus values varied from -1.5 to -1.7 μm. Image stacks were acquired with an exposure time of 3.8 s and fractionated into 32 frames with a total dose of 50 $e^{-}$ Å$^{-2}$. The stacks were motion corrected with MotionCor2[1] and binned two-fold, resulting in a pixel size of 0.82 Å/pixel; meanwhile, dose weighting was performed[2]. The defocus values were estimated via patch CTF estimation.

### Cryo-EM data processing

In total, 1,630, 1,434, 2,731, and 1,799 micrographs were collected for CqsS, CqsS-ATPγS, CqsS-CqsA-CAI-1, and CqsS-CAI-1 (no CqsA), respectively. After the particles were picked, 1,492,169, 1,523,166, 1,679,523, and 2,163,879 particles were extracted for further data processing, respectively. According to the micrographs of the CqsS-CqsA-CAI-1 complex, two EM structures corresponding to the ICD and TMD were determined, which were designated CqsS-CqsA (ICD structure) and CqsS-CAI-1 (TMD structure) in the main text.

[CqsS]

The particles were applied to 2D classification, resulting in 188,821 good particles. After ab-initio reconstruction with 4 classes, one good class was outstanding. This good class served as reference for heterogeneous refinement, yielding good particles for another run of ab-initio reconstruction. The resulting good class, containing 271,340 particles, was selected as seeds for seed-facilitated classification to select additional good particles from the original particles, resulting in 713,012 good particles. These good particles were further subjected to heterogeneous refinement, resulting in 356,995 good particles and a 3.08 Å 3D reconstruction. After 3D classification with a mask on the ICD, two distinct classes were selected, Class A with 124,184 particles and Class B with 137,177 particles, yielding 3D reconstitutions of 3.24 Å and 3.28 Å, respectively. These two classes were designated $CqsS_{S1}$ and $CqsS_{S2}$, respectively.

[CqsS-ATPγS]

After 2D classification, a total of 381,945 good particles were selected and subjected to ab-initio reconstruction, resulting in a good class with 227,283 particles. These particles were selected as seeds for seed-facilitated classification, yielding 580,605 good particles. After a further 3D classification with a mask on the ICD, two distinct classes were selected, Class A with 150,052 particles and Class B with 140,065 particles, yielding 3D reconstitutions with 3.48 Å and 3.46 Å, respectively. These two reconstructions were designated CqsS-ATPγ$S_{S1}$ and CqsS-ATPγ$S_{S2}$, respectively.

[CqsS-CqsA-CAI-1]

After 2D classification, 2D averages could be separated into two major classes: averages with stronger densities in the ICD and those with stronger densities in the TMD. These two types of 2D averages were chosen as template 1 and template 2 to select additional particles from the original micrographs with estimated particle diameters of 170 Å and 120 Å, resulting in 1,855,902, and 3,415,200 particles, respectively. These particles were applied to the structural determination of the ICD and TMD of this complex.

For the ICD, 1,855,902 particles were subjected to several rounds of 2D classification, resulting in a total of 318,201 good particles. These particles were used in ab-initio reconstruction, resulting in good reconstruction with 200,271 particles. These particles were selected as seeds for seed-facilitated classification, yielding 470,547 good particles. After further 3D classification, the ICD of CqsS-CqsA was determined at 2.87

Å resolution with 130,780 particles. This structure is designated CqsS-CqsA in the main text.

For the TMD, 3,415,200 particles were subjected to 2D classification, heterogeneous refinement, and ab initio reconstruction, resulting in a good reconstruction with 239,375 particles. These particles were selected as seeds for seed-facilitated classification, yielding 737,497 good particles. After several rounds of heterogeneous refinement and 3D classification, 131,035 good particles were selected which yielded a 3D reconstruction at 2.87 Å resolution after non-uniform refinement with a mask on the TMD. This structure is referred to as CqsS-CAI-1 in the main text.

[CqsS-CAI-1 (no CqsA)]

After 2D classification and removal of particles with preferred orientations, 891,414 particles were selected and subjected to heterogeneous refinement, yielding a good class containing 341,471 particles. These particles were next subjected to two rounds of ab initio reconstructions, resulting in 68,546 good particles. These particles generated a final reconstruction at 6.5 Å resolution after non-uniform refinement.

**Model building and refinement**

The structure of CqsS was built primarily based on the $CqsS_{S1}$ map at 3.24 Å resolution. AlphaFold2-predicted models[3] facilitated model building for regions with moderate

resolution. The resulting structure served as an initial reference for model building of $CqsS_{S2}$, CqsS-ATPγ$S_{S1}$, CqsS-ATPγ$S_{S2}$, CqsS-CqsA, and CqsS-CAI-1. Manual adjustment was made in Coot[4], and all structure refinement was carried out by PHENIX[5] in real space with secondary structure and geometry restraints. All the structure figures were prepared with PyMol[6], and all the EM maps were prepared with Chimera[7].

### *In vitro* CqsS phosphorylation assay

Phosphorylation assays were conducted in phosphorylation buffer (50 mM Tris pH 8.0, 100 mM KCl, 5 mM $MgCl_2$, and 10% (v/v) glycerol) with 2 μM CqsS, CqsS mutants, or CqsS chimeras. For the assays involving LuxU, LuxU was added at 10 μM. For the CqsA inhibition experiments, 2 μM CqsA was included. For the CAI-1 inhibition experiments, CAI-1 was produced by overexpressing CqsA in *E. coli* BL21 cultured in LB medium as previously reported [8], and cell-free fluid was added to the assay system 30 min before the reactions were initiated. Regarding the DAG inhibition experiments, DAG (1,2-dipalmitoyl-sn-glycerol, 16:0/16:0, Sigma-Aldrich) and POPC (Anatrace) were individually dissolved in chloroform and combined at an ~1:1 molar ratio. The solution was evaporated to dryness under a gentle nitrogen stream. The dried film was subsequently resuspended in 25 mM Tris pH 8.0, 150 mM NaCl, 2 mM sodium deoxycholate (Aladdin), yielding a final DAG concentration of 10 mM. The suspension was sonicated for 5 min and diluted into the reaction mixture at the indicated final concentrations. All experiments were initiated by adding 100 μM cold ATP and 0.5 μCi

[γ-$^{32}$P]-ATP (from a stock of 3,000 Ci/mmol, Revvity). To verify *cis* phosphorylation on CqsS H1, only 0.5 μCi [γ-$^{32}$P]-ATP was used to initiate the reaction, and no cold ATP was added. After incubation at room temperature for 10 min, the reactions were terminated with SDS loading buffer. Following separation via SDS-PAGE, gels were exposed to a phosphoscreen overnight and subsequently analyzed via Amersham™ Typhoon™ IP and ImageJ.

***In vivo* bioluminescence assay**

WT *V. harveyi* N8T11 *cqsA* and *cqsS* sequences were subcloned into pMMB, together with the corresponding *cqsAS* upstream and downstream regulatory regions from *V. harveyi* BB120. This construct was used as the template to generate the desired *cqsS* mutations via site-directed mutagenesis. To test the effects of mutations on CqsS function, the different versions of *cqsAS* along with the upstream and downstream *V. harveyi* BB120 regulatory DNA were amplified via PCR and inserted into the pLAFR2 cosmid via the BamHI and XbaI restriction sites. The resulting constructs were introduced into *V. harveyi* WN1397 (BB120 Δ*luxN* Δ*luxPQ* Δ*cqsAS*) by conjugation. Exconjugate strains were grown overnight in LM at 30°C with shaking, followed by back-dilution to $OD_{600}$ = 0.005. Aliquots (150 μL) of the cultures were transferred into the wells of 96-well plates (Corning). The plates were incubated at 30°C (Biotek Biospa). The plates were shaken every 15 min. Light production and $OD_{600}$ were measured (Biotek Neo). Regarding the DAG inhibition experiment, bioluminescence output from WT Δ*luxN* Δ*luxPQ* Δ*cqsA* *V. harveyi* were measured. Strains were grown

overnight in LM at 30°C with shaking, followed by back-dilution to $OD_{600}$ = 0.005. Aliquots (150 μL) of the cultures were transferred into the wells of 96-well plates (Corning). All well were administered 50 μL of cell-free culture fluid as designated in the figure and either 250 μM 16:0 1,2-dipalmitoyl-sn-glycerol (Avanti Research) or ethanol. The plates were incubated at 30°C with continuous shaking. Light production and $OD_{600}$ were measured every 10 min (Biotek Synergy). For all experiments, light output is reported as relative light units (RLUs), which is the bioluminescence/$OD_{600}$.

**CqsS-CqsA pull-down assay**

0.2 mg of His-tagged CqsA and 0.1 mg of Strep-tagged CqsS were incubated in 1 mL of buffer A on ice for 1 h. Subsequently, the mixtures were loaded onto 300 μL of Strep-Tactin® Sepharose®. After washing with buffer A, the resin was eluted with buffer A supplemented with 2.5 mM desthiobiotin. Eluates were subjected to SDS–PAGE analysis. To evaluate the effect of CAI-1 on the interaction, cell-free culture fluids from *E. coli* expressing CqsA or the inactive CqsA K240A variant were included during the incubation period.

**Statistics and reproducibility**

No statistical methods were used to predetermine sample sizes. The experiments were not randomized, and the investigators were not blinded during the experiments nor outcome assessments. Each experiment was conducted independently at least three times with similar results.

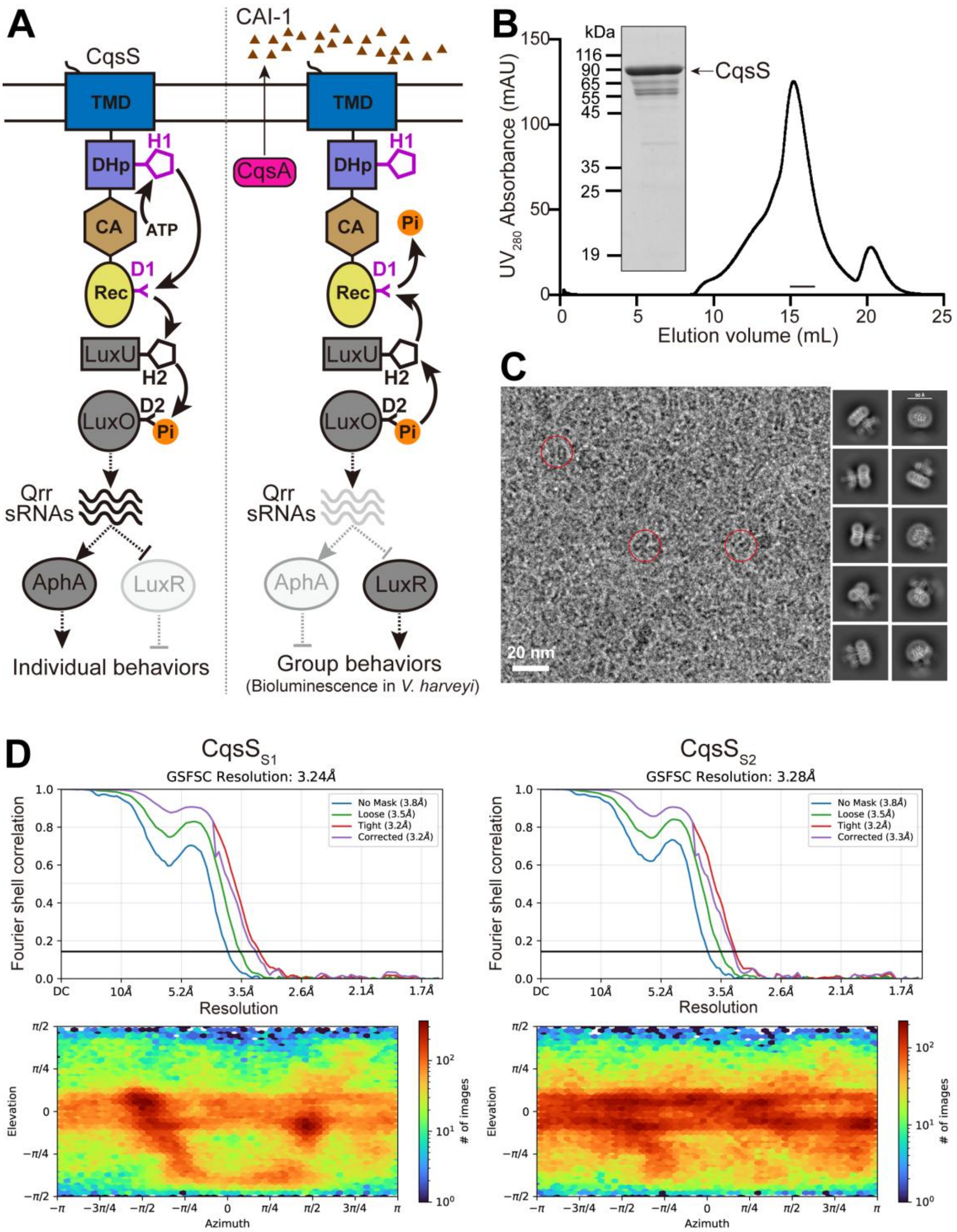


**Fig. S1. Cryo-EM analysis of *V. harveyi* CqsS**. (*A*) A schematic diagram illustrating CqsS-mediated QS signal transduction under low (*left*) and high (*right*) cell density conditions in vibrios. The brown triangles represent the CAI-1 autoinducer. (*B*) Representative size exclusion chromatography (SEC) profile of CqsS solubilized in

0.005% LMNG. Peak fractions were concentrated for cryo-EM sample preparation. *Inset*: SDS-PAGE analysis of the concentrated CqsS protein used for cryo-EM sample preparation. The experiments were independently repeated more than three times with similar results. (*C*) Representative micrographs (*left*) and 2D class averages (*right*) of cryo-samples of CqsS. The representative particles are indicated by red circles. Scale bar, 20 nm. (*D*) Golden standard Fourier shell correlation (GSFSC) curves (*top*) and angular distribution maps (*bottom*) for cryo-EM reconstructions of $CqsS_{S1}$ (*left*) and $CqsS_{S2}$ (*right*).

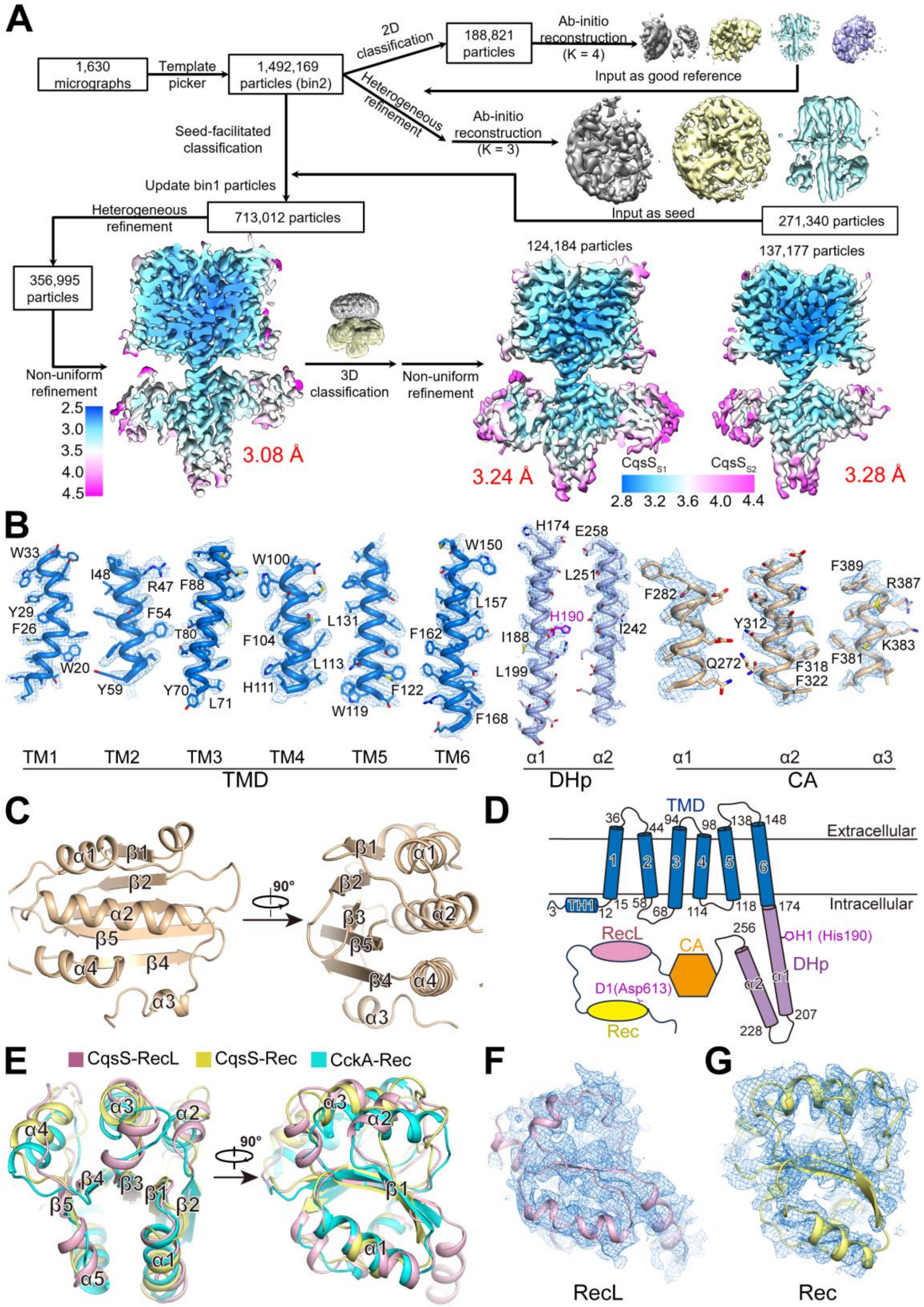


**Fig. S2. Structural determination and model building of** CqsS. (*A*) Flowchart for the structural determination of CqsS in two states, $CqsS_{S1}$ and $CqsS_{S2}$. The unit of resolution is Å. Details are provided in *Materials and Methods*. (*B*) EM maps of

representative structural elements. The densities, contoured at 5-6σ, were prepared in PyMOL based on the $CqsS_{S1}$ map. (*C*) The structure of the CqsS CA domain. This domain consists of 4 helices and 5 sheets. (*D*) Membrane topology of CqsS based on the cryo-EM structures. (*E*) Structural overlay of the RecL and Rec domains of CqsS relative to the Rec domain from the representative hybrid histidine kinase CckA (PDB code: 6TNE). (*F*-*G*) EM maps of the RecL (*F*) and Rec (*G*) domains of CqsS. All figures were generated based on the CqsS-A protomer in the $CqsS_{S1}$ structure.

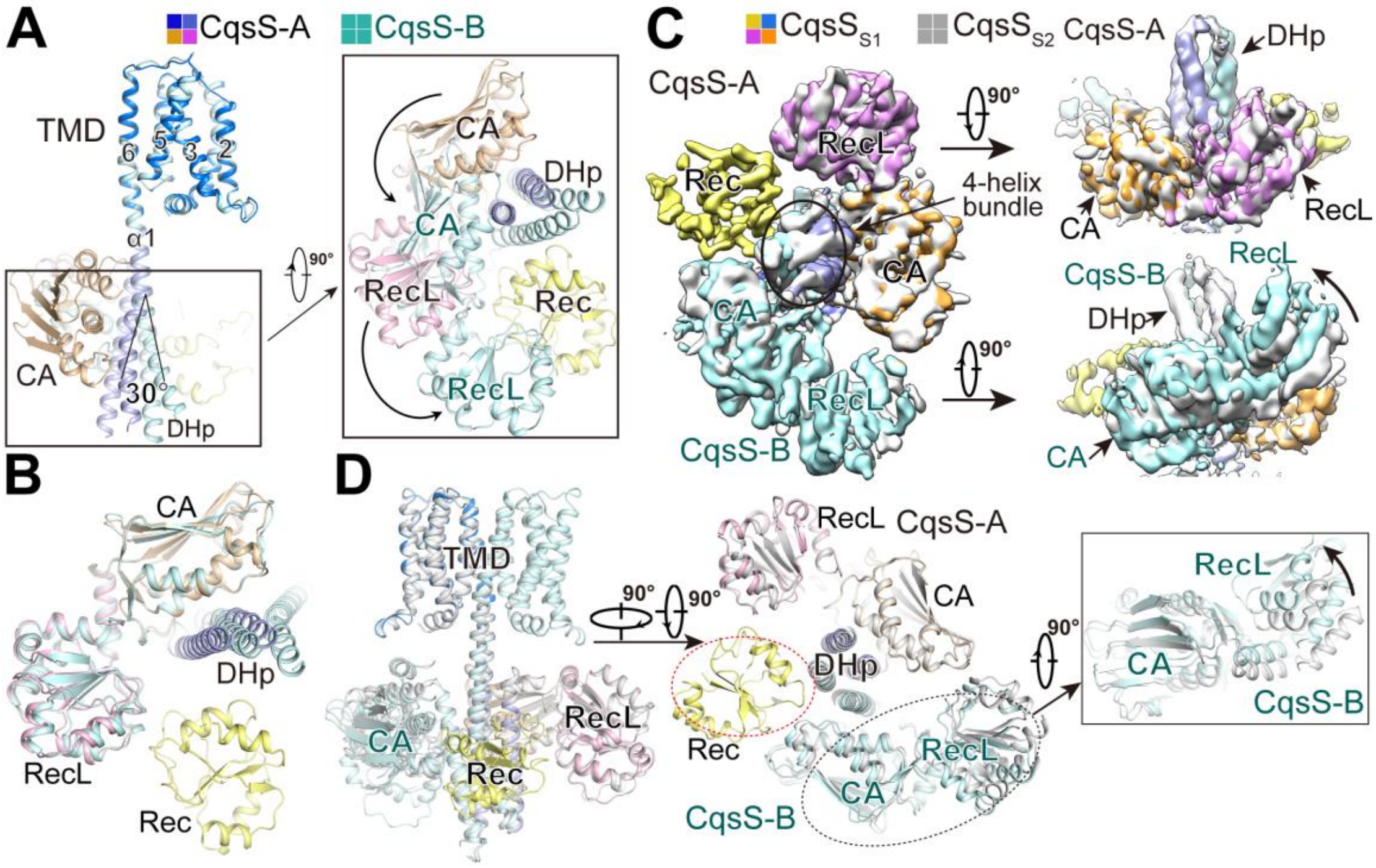


**Fig. S3. Structural comparison of $CqsS_{S1}$ and $CqsS_{S2}$.** (*A*) Structural superimposition of two protomers (CqsS-A and CqsS-B) in the $CqsS_{S1}$ structure relative to their TMDs. *Inset*: Zoomed-in view highlighting structural differences in the cytosolic domains between the two protomers. (*B*) A cytosolic view showing structural comparison between the CqsS-A and CqsS-B protomers relative to their CA domains. (*C*) Superimposition of the EM maps of $CqsS_{S1}$ and $CqsS_{S2}$ to reveal differences in their cytosolic domains (*left*), and two separate views (*right*) show the differences in each protomer: CqsS-A (*top*) and CqsS-B (*bottom*). (*D*) One membrane view and one extracellular view to compare the $CqsS_{S1}$ and $CqsS_{S2}$ structures. The Rec domain of CqsS-A is not visible in the $CqsS_{S2}$ structure, as indicated by the red ellipse, and obvious structural differences are highlighted in the CA and RecL domains of CqsS-B by the black ellipse. *Inset*: Zoomed-in view showing the conformational changes that exist in the CA and RecL domains of CqsS-B between these two structures.

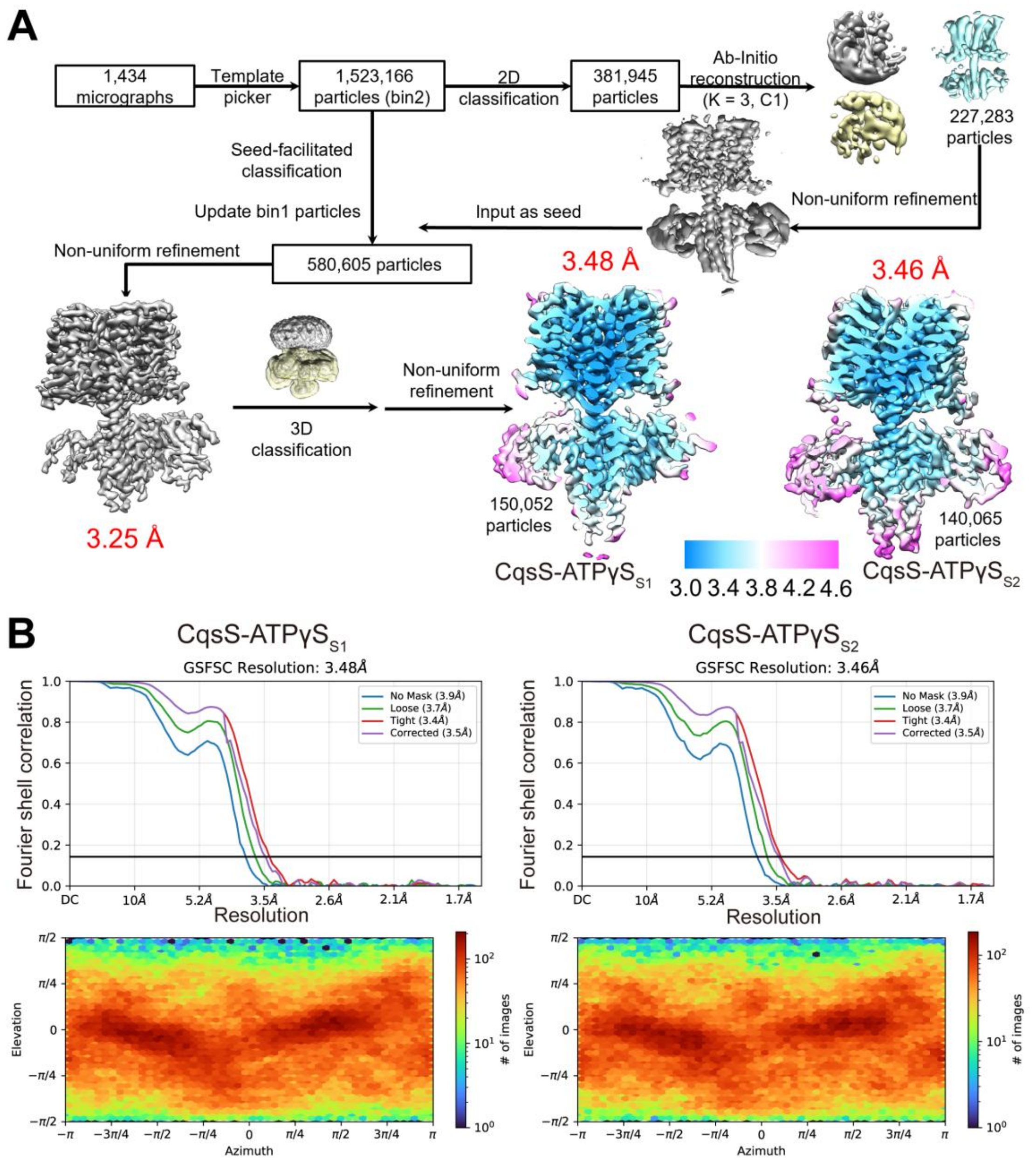


**Fig. S4. Structure determination of CqsS in the ATPγS-bound state.** (*A*) Flowchart for the structural determination of CqsS-ATPγS complexes in two states, CqsS-ATPγS$_{S1}$ and CqsS-ATPγS$_{S2}$. The unit used for resolution is Å. (*B*) GSFSC curves (*top*) and angular distribution maps (*bottom*) for CqsS-ATPγS$_{S1}$ (*left*) and CqsS-ATPγS$_{S2}$ (*right*).

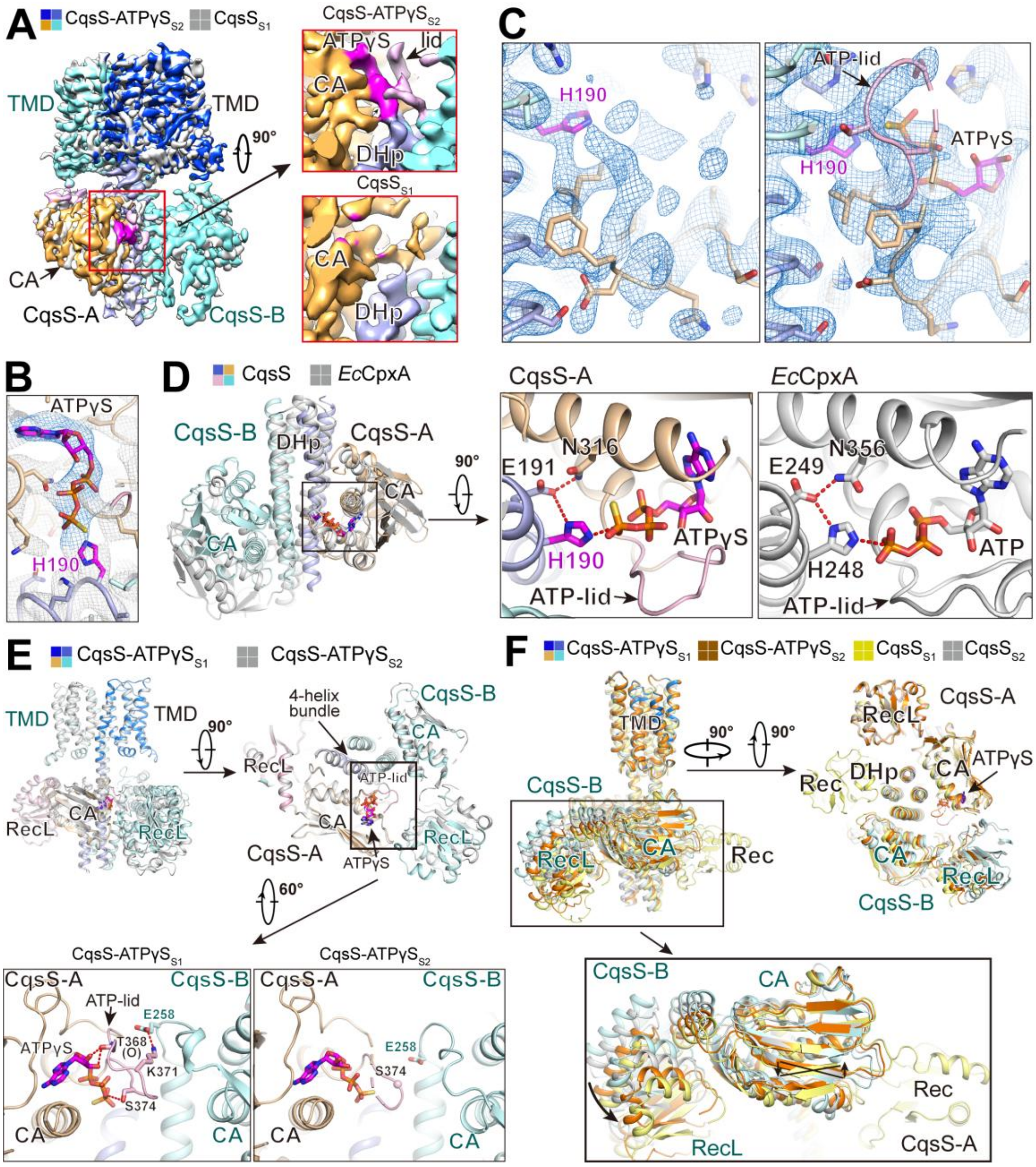


**Fig. S5. Structural comparison of CqsS in the apo- and ATPγS-bound states.** (*A*) Overlay of maps between $CqsS_{S1}$ and $CqsS\text{-}ATP\gamma S_{S2}$. *Insets*: Zoomed in views showing the ATP binding site in the CA domain of the CqsS-A protomer in $CqsS\text{-}ATP\gamma S_{S2}$ (*top*) and $CqsS_{S1}$ (*bottom*). (*B*) Structural modeling of the ATPγS molecule into the densities in the $CqsS\text{-}ATP\gamma S_{S2}$ structure. (*C*) Comparative views of EM maps at the ATP-lid region between $CqsS_{S1}$ (*left*) and $CqsS\text{-}ATP\gamma S_{S2}$ (*right*). The EM maps for this region

of CqsS-ATPγ$S_{S1}$ are shown in Fig. 2*C*. All the EM maps are contoured at 6σ. (*D*) Structural comparison between the histidine kinase domains (DHp and CA domains) of CqsS and the classic histidine kinase domain of *E. coli* CpxA (PDB code, 5LFK). *Insets*: Zoomed-in views detailing the interactions between the CA and DHp domains in these two proteins. (*E*) Structural comparison between CqsS-ATPγ$S_{S1}$ and CqsS-ATPγ$S_{S2}$. *Top*: A membrane view (*left*) and an extracellular view (*right*) are presented to show the overlay of the two structures. *Bottom*: Zoomed in views showing the interaction between two CA domains in the two structures. A salt bridge between CqsS-A Lys371 and CqsS-B Glu258 is observed in the CqsS-ATPγ$S_{S1}$ structure (*left*) but not in the CqsS-ATPγ$S_{S2}$ structure (*right*), potentially explaining the partial visualization of the ATP-lid loop in CqsS-ATPγ$S_{S2}$. (*F*) Structural comparison between CqsS$_{S1}$, CqsS$_{S2}$, CqsS-ATPγ$S_{S1}$, and CqsS-ATPγ$S_{S2}$. *Top*: One membrane (*left*) and one cytosolic (*right*) view showing structural overlays of these four structures. The TMDs are nearly identical, while some obvious structural differences are observed in the cytosolic domains, primarily in the CqsS-B protomer. *Bottom*: Zoomed in view showing the conformational changes in the CA and RecL domains of CqsS-B among these structures.

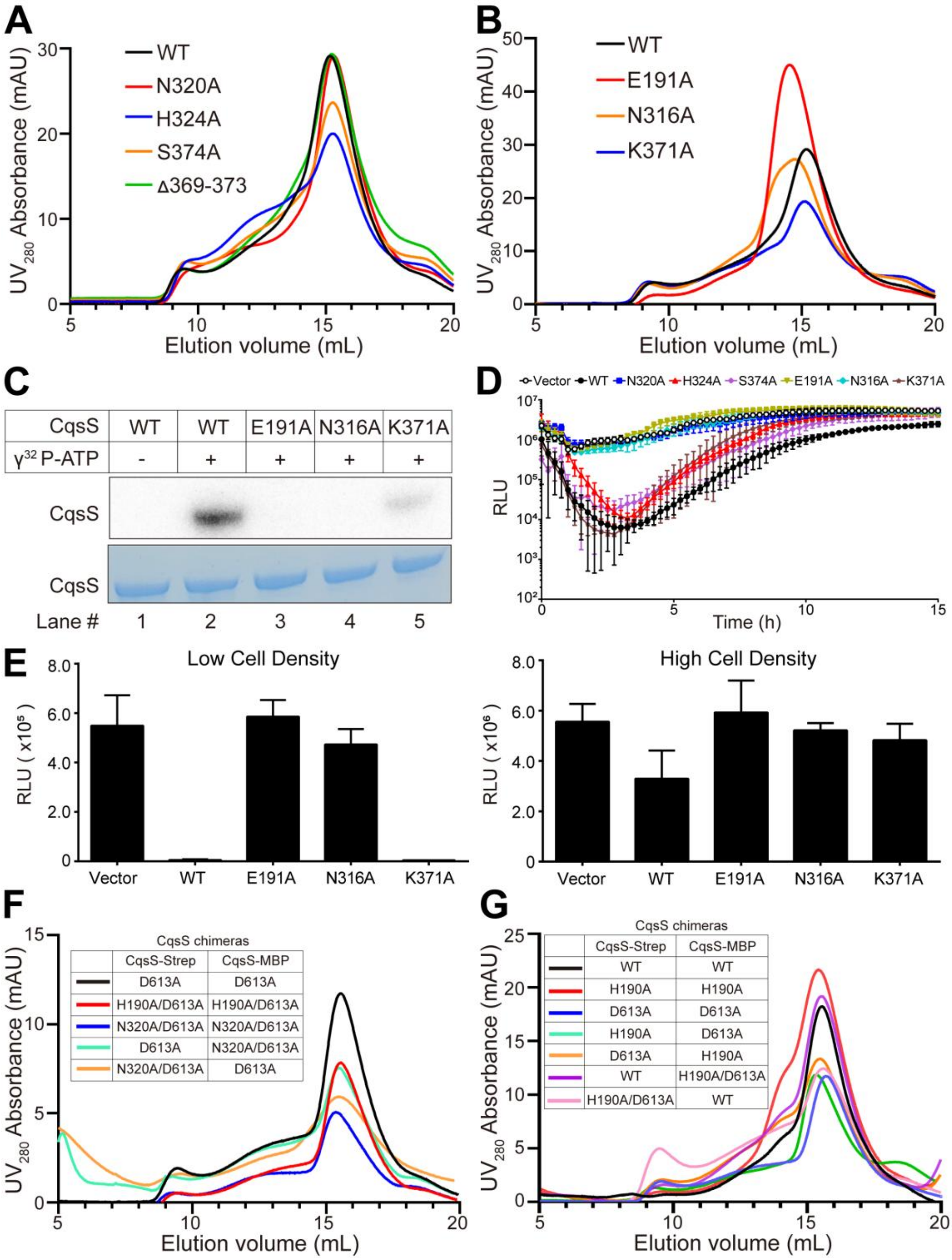


**Fig. S6. SEC profiles of WT CqsS, CqsS variants, and CqsS chimeras used in phosphorylation assays.** (*A*) SEC profiles of WT CqsS and CqsS variants investigated for ATPγS coordination. (*B*) SEC profiles of WT CqsS and CqsS variants investigated for interactions between the CA and DHp domains. The CqsS variants in panels (*A*) and

(*B*) have elution volumes comparable to that of the WT CqsS, supporting their use in the *in vitro* autophosphorylation assays shown in Fig. 2*F* and *SI Appendix*, Fig. S6*C*. (*C*) Validation of the essential residues involved in the CqsS CA-DHp interaction as judged by the CqsS autophosphorylation assay. Purified WT and variant CqsS proteins were incubated with radioactive ATP ($\gamma^{32}$P-ATP) in phosphorylation buffer, followed by autoradiography (*top*, gray) and Coomassie blue staining (*bottom*, blue). (*D*) The bioluminescence output of *V. harveyi* containing the designated *cqsS* mutants was assessed continuously over time. The minimum and maximum RLU values were selected to generate the bar graphs for low and high cell densities presented in Fig. 2*G* and *SI Appendix*, Fig. S6*E*, respectively. See *Materials and Methods* for all assay conditions. (*E*) Validation of the essentiality of the designated residues involved in intra-domain interactions via assessment of *in vivo* kinase activity in *V. harveyi* carrying different CqsS variants. The graphs present the minimum and maximum light output in the assay, which occur at low cell density (*left*) and high cell density (*right*), respectively. In panels (*D*) and (*E*), RLU refers to the relative light units, which are the bioluminescence per $OD_{600}$. Error bars denote standard deviations of the means, *n*=3. (*F*-*G*) SEC profiles of CqsS-Strep and CqsS-MBP chimeras used to verify *cis* phosphorylation on the CqsS H1 residue (*F*) and *trans* phospho-transfer from the H1 to D1 residue (*G*). The mutations present in each subunit of the CqsS chimeras are detailed in the accompanying tables. See *Materials and Methods* for details.

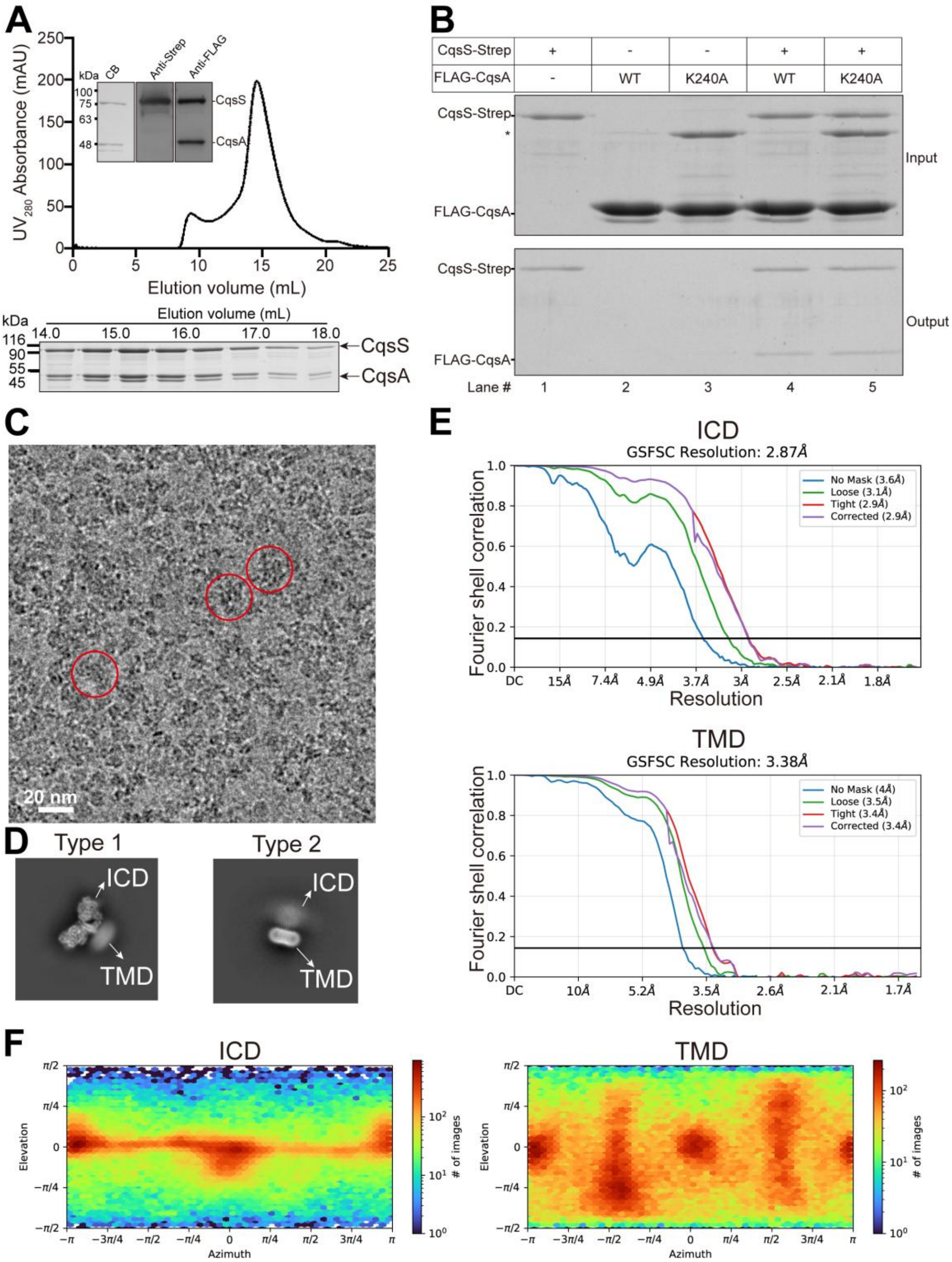


**Fig. S7. Cryo-EM analysis of the CqsS-CqsA-CAI-1 complex.** (*A*) Representative SEC profile of the CqsS-CqsA-CAI-1 complex. *Insets*: SDS-PAGE analysis of the peak fractions, followed by Coomassie blue analysis (*left*) and immunoblotting analyses

using anti-Strep (*middle*) and anti-FLAG (*right*) antibodies. The peak fractions were concentrated for cryo-EM sample preparation (*bottom*). The experiments were independently repeated more than three times with similar results. (*B*) The catalytically-inactive CqsA K240A mutant protein interacts with CqsS with comparable avidity as WT CqsA. WT CqsA and CqsA K240A were incubated with CqsS followed by affinity purification using a Strep tag on CqsS. See *Materials and Methods* for details. (*C*) Representative micrograph of a cryo-EM sample of the CqsS-CqsA-CAI-1 complex. The representative particles are indicated by red circles. Scale bar, 20 nm. (*D*) Typical images of two distinct types of 2D averages. *Left*: 2D average with a clear intracellular domain (ICD) but a blurred TMD; *right*: 2D average with a clear TMD but a blurred ICD. (*E*) GSFSC curves for the final reconstructions of the ICD (*top*, CqsS-CqsA) and TMD (*bottom*, CqsS-CAI-1) for the CqsS-CqsA complex. (*F*), Angular distribution maps for the final reconstructions of the ICD (*left*, CqsS-CqsA) and TMD (*right*, CqsS-CAI-1) for the CqsS-CqsA complex. The ICD and TMD structures are referred to as the CqsS-CqsA and CqsS-CAI-1 structures, respectively, in the main text.

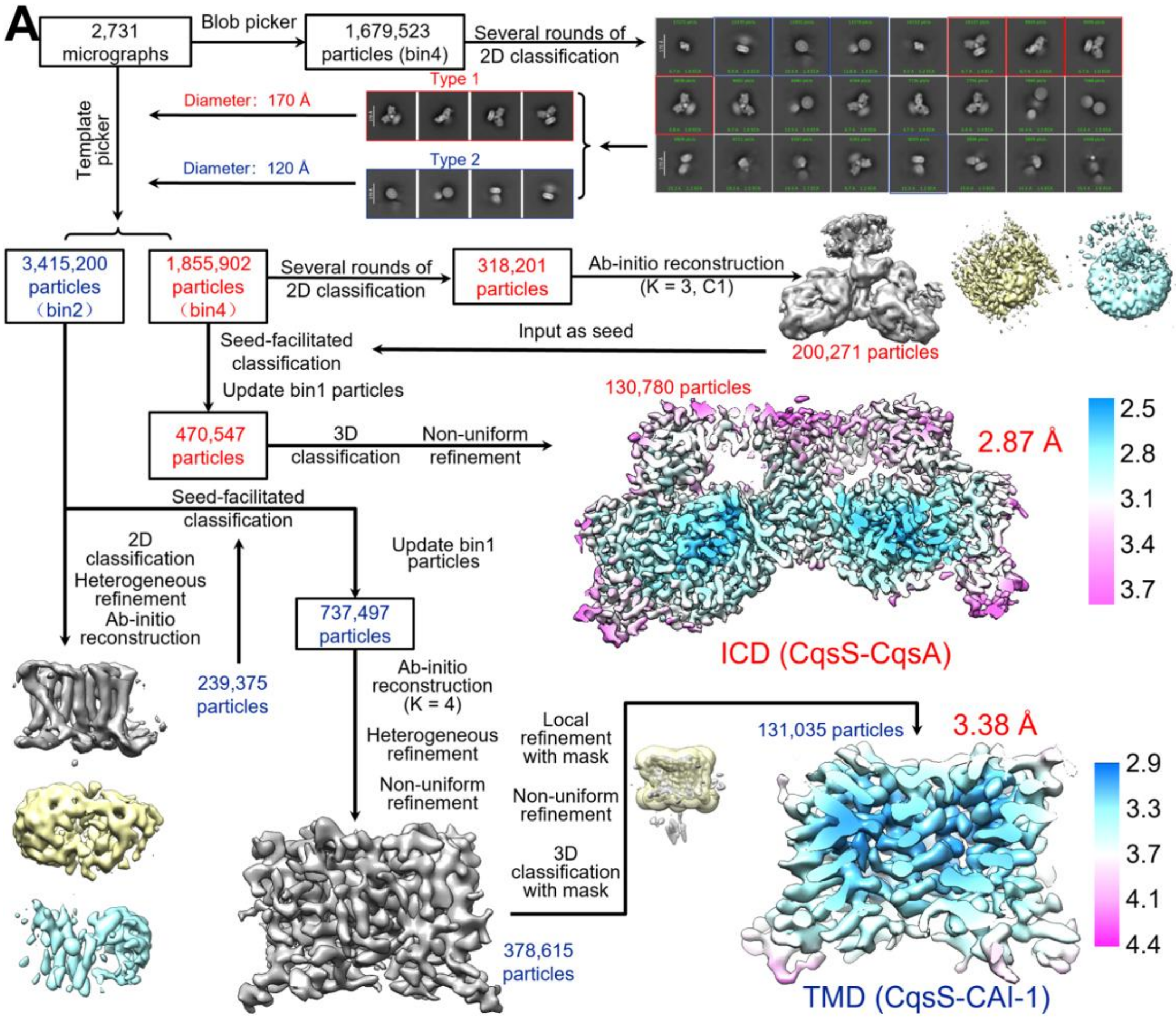


**Fig. S8. Structural determination of the CqsS-CqsA-CAI-1 complex.** Flowchart for the structural determination of the ICD and TMD of the CqsS-CqsA-CAI-1 complex. The unit used for resolution is Å. Red and blue text indicate the structural determination of the ICD and TMD, which are referred to as the CqsS-CqsA and CqsS-CAI-1 structures in the main text, respectively. See *Materials and Methods* for details.

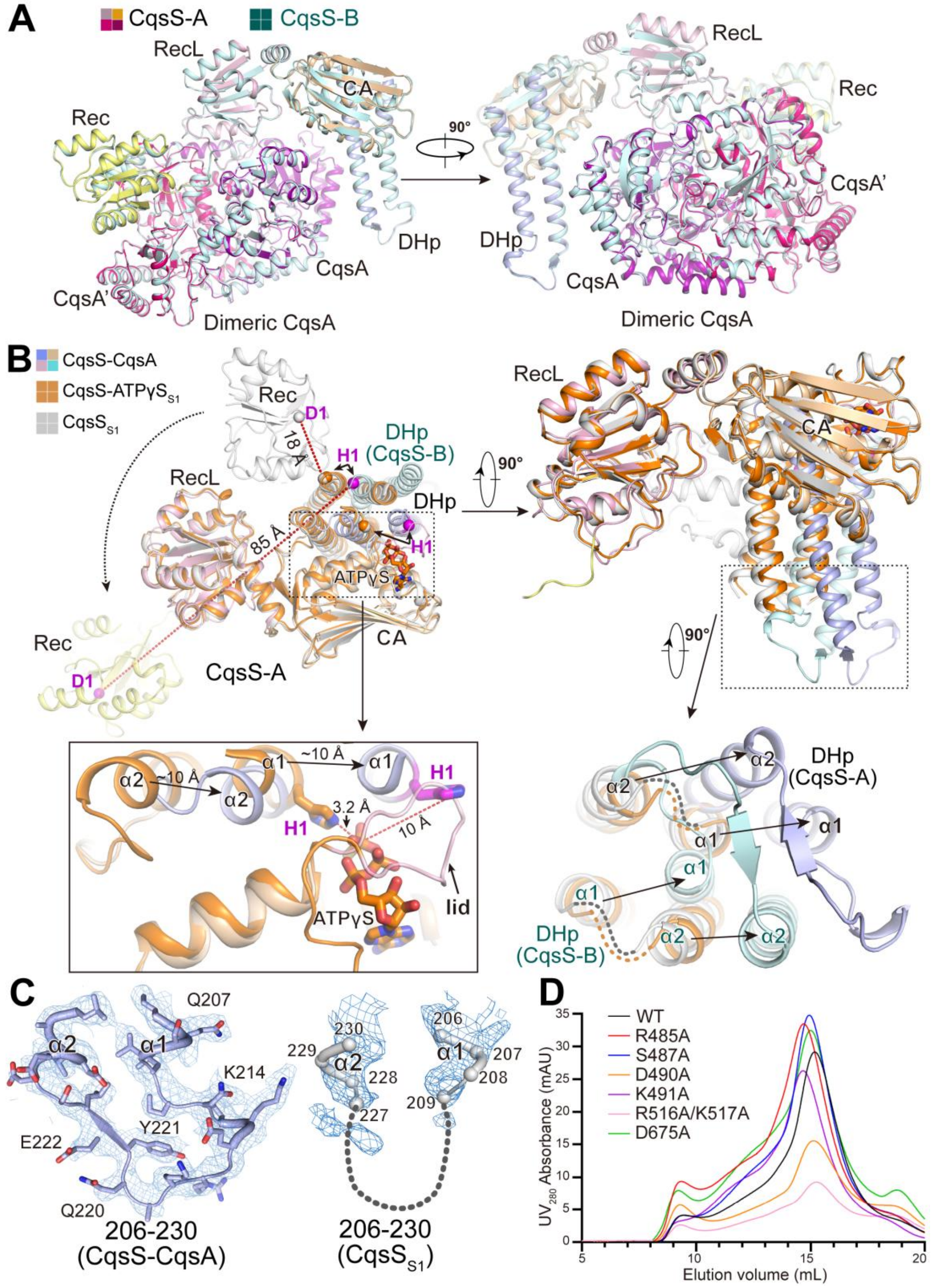


**Fig. S9. Structural comparison among CqsS-CqsA, CqsS-ATPγS$_{S1}$, and CqsS$_{S1}$.**

(*A*) Two views showing superimpositions of two 1:2 CqsS-CqsA subcomplexes,

suggesting high similarity. (*B*) Structural overlay of the CqsS-A protomer in the CqsS-CqsA, CqsS-ATPγ$S_{S1}$, and Cqs$S_{S1}$ structures. The DHp domain of CqsS-B is included to illustrate the distance between the H1 residue of CqsS-B and the D1 residue of CqsS-A, where a *trans* phospho-transfer may occur. *Top*: Two views displaying the structural superimposition of the cytosolic domains of CqsS-A in relation to the CA and RecL domains for these structures. *Bottom*: Zoomed-in views showing the ATP binding pocket within the CA domain (*left*) and highlighting the structural differences between the DHp domains in these structures (*right*). (*C*) EM maps for the loops (residues 206-230) connecting the α1 and α2 helices of the DHp domains in the CqsS-CqsA complex (*left*) and Cqs$S_{S1}$ (*right*) structures. In the CqsS-CqsA complex, this loop is well-resolved, whereas it is poorly resolved in the Cqs$S_{S1}$ structure. The EM maps are contoured at 5σ. (*D*) SEC profiles of CqsS mutants with substitutions in residues involved in interaction with CqsA. The CqsS variants have elution volumes similar to that of the WT CqsS.

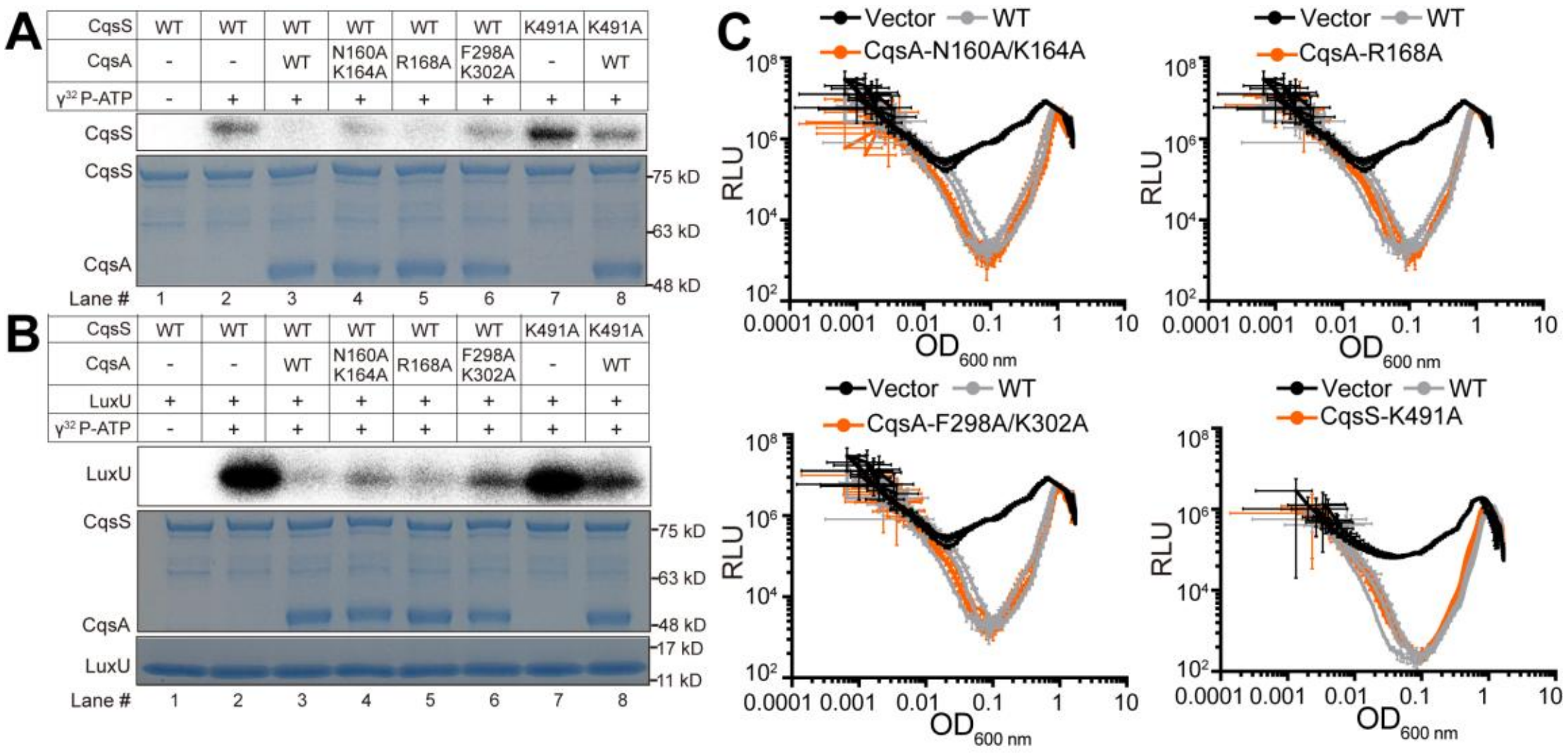


**Fig. S10. Functional evaluation of representative CqsA and CqsS mutants with impaired CqsS-CqsA interactions.** (*A*) CqsA-mediated inhibition of CqsS autophosphorylation by the designated mutants. Purified CqsS proteins were incubated with radioactive ATP ($\gamma^{32}$P-ATP) and CqsA proteins in phosphorylation buffer, followed by autoradiography (*top*, gray) and Coomassie blue staining (*bottom*, blue). (*B*) CqsA-mediated inhibition of phospho-relay to LuxU by the designated mutants. CqsA proteins were incubated with purified CqsS protein, radioactive ATP ($\gamma^{32}$P-ATP), and LuxU in phosphorylation buffer, followed by autoradiography (*top*, gray) and Coomassie blue staining (*bottom*, blue). (*C*) Bioluminescence output was assessed over growth for *V. harveyi* strains carrying the *cqsA* N160A/K164A, R168A, F298A/K302A, and *cqsS* K491A variants.

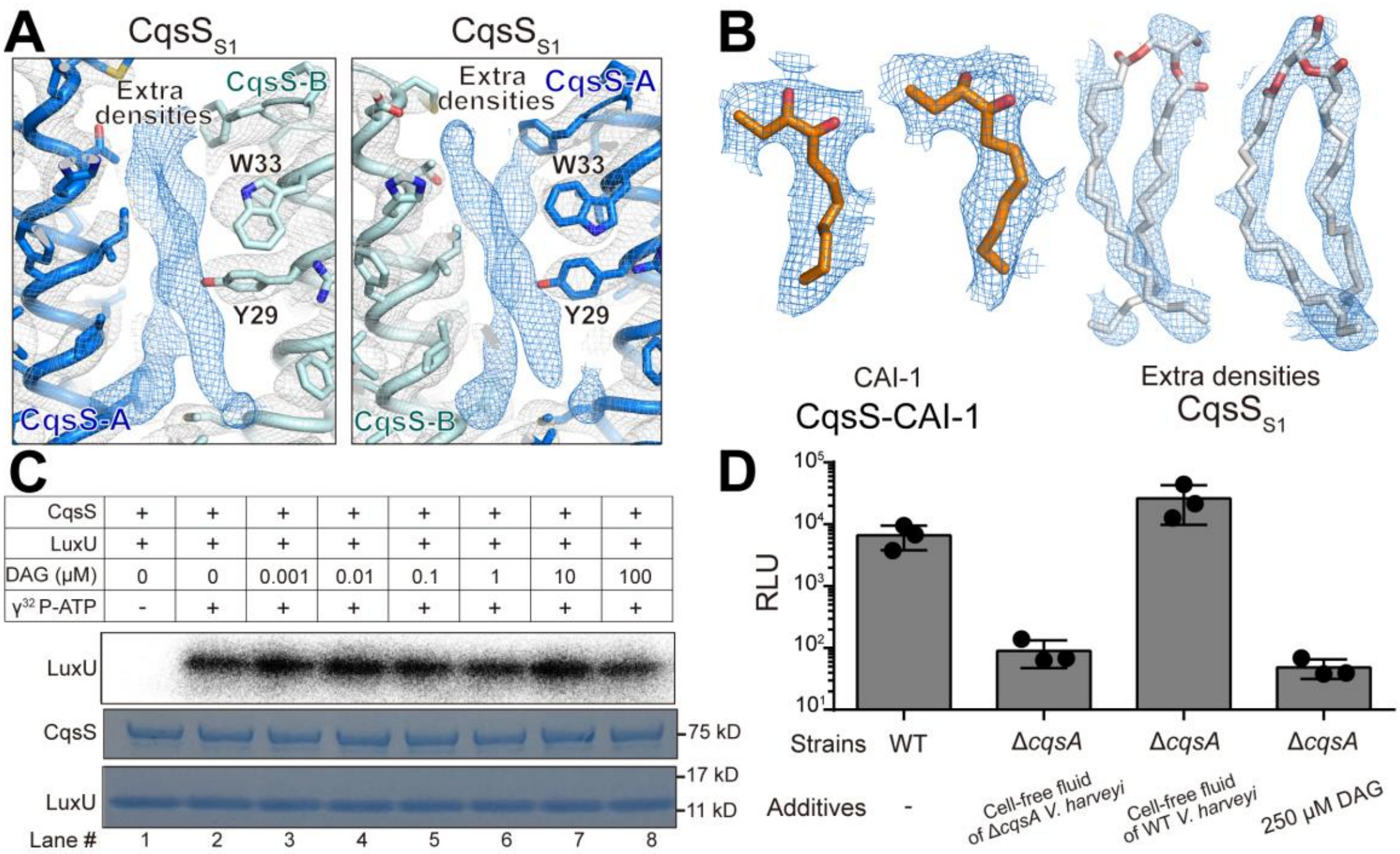


**Fig. S11. Additional densities at the CAI-1 binding sites in the CqsS$_{S1}$ structure.**

(*A*) The extra densities at the CAI-1 binding sites in the CqsS$_{S1}$ structure are contoured at 6σ. For comparison, please refer to Fig. 6*C* for the CAI-1 densities in the CqsS-CAI-1 complex. (*B*) EM maps for the CAI-1 molecules in CqsS-CAI-1 complex (*left*) and the extra densities at the corresponding sites in CqsS$_{S1}$ (*right*) shown with diacylglycerol (DAG) modeled as the compound in the structure. The EM maps are contoured at 6σ. (*C*) Phospho-relay from CqsS to LuxU in the presence of the indicated concentrations of DAG. (*D*) CqsA-CqsS-driven bioluminescence output by the designated *V. harveyi* strains in response to the indicated conditions. The DAG used is dipalmitoyl glycerol.

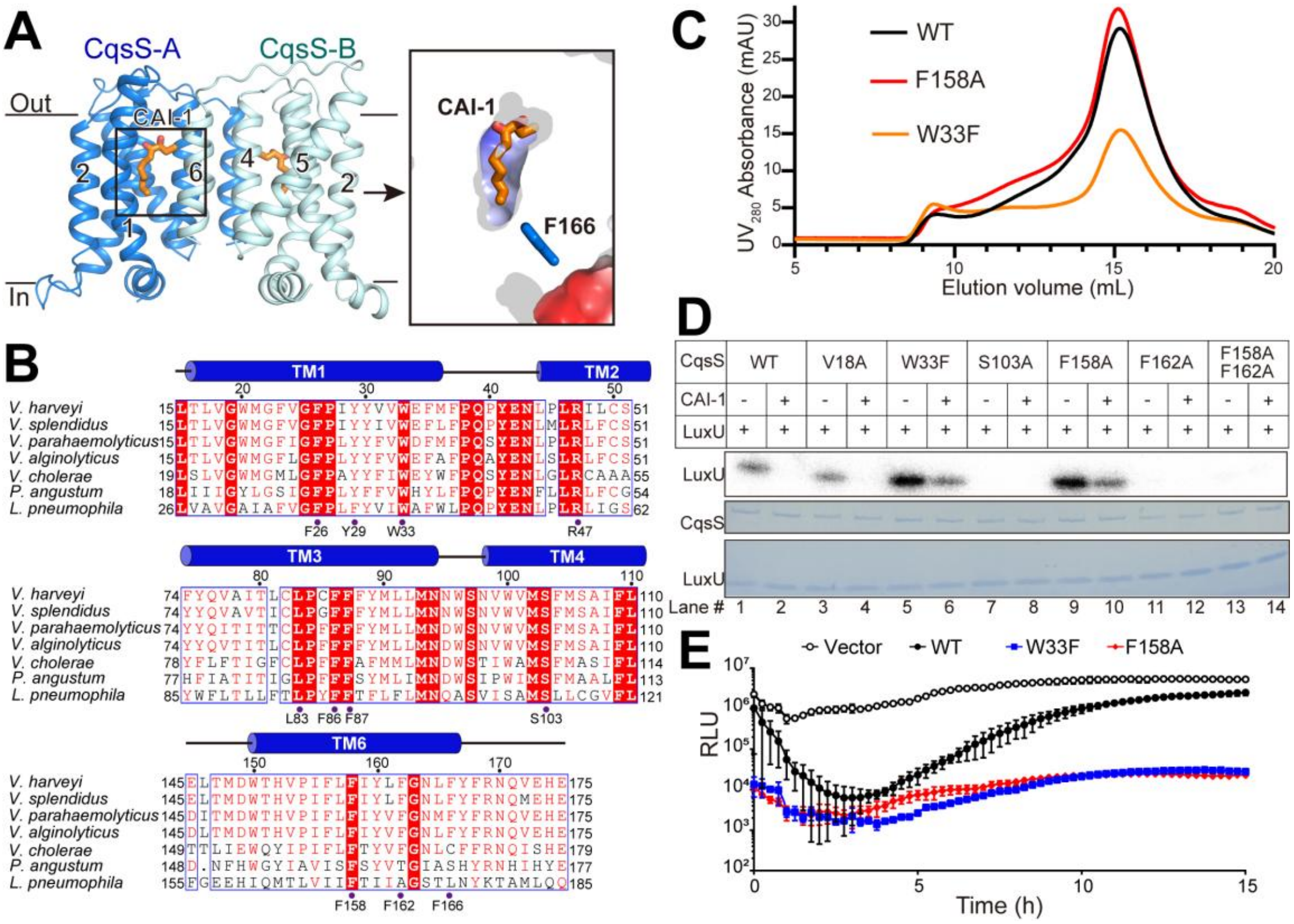

**Fig. S12. Validation of essential residues involved in CAI-1 coordination and signal transduction.** (*A*) Sectional view of the CAI-1 binding pocket in the CqsS-A protomer. Phe166 is located at the bottom of the ligand binding pocket and restricts the available space. (*B*) CqsS sequence alignment. The highly conserved CAI-1-coordinating residues are indicated with purple dots below the alignment. *V.*, *Vibrio*; *P. angustum*, *Photobacterium angustum*; *L. pneumophila*, *Legionella pneumophila*. (*C*) SEC profiles of WT CqsS and the designated variants used to validate the residues that participate in CAI-1 coordination. The CqsS variants have elution volumes similar to that of the WT CqsS. (*D*) *In vitro* assessment of the kinase activities of WT CqsS and

the designated CqsS variants with substitutions in residues involved in CAI-1 coordination. Purified CqsS proteins were incubated with radioactive ATP ($\gamma^{32}$P-ATP) and purified LuxU protein in the presence or absence of 5% cell-free culture fluid containing CAI-1, followed by autoradiography (*top*, gray) and Coomassie blue staining (*bottom*, blue). (*E*) Bioluminescence output of Δ*luxN* Δ*luxPQ* Δ*cqsS V. harveyi* was assessed continuously over time following the introduction of *cqsS* W33F and *cqsS* F158A. RLU denotes relative light units, which are bioluminescence per $OD_{600}$. Error bars denote standard deviations of the means, *n*=3. The minimum and maximum RLU values were selected to generate the bar graphs for low and high cell densities, respectively, in Fig. 7*D*.

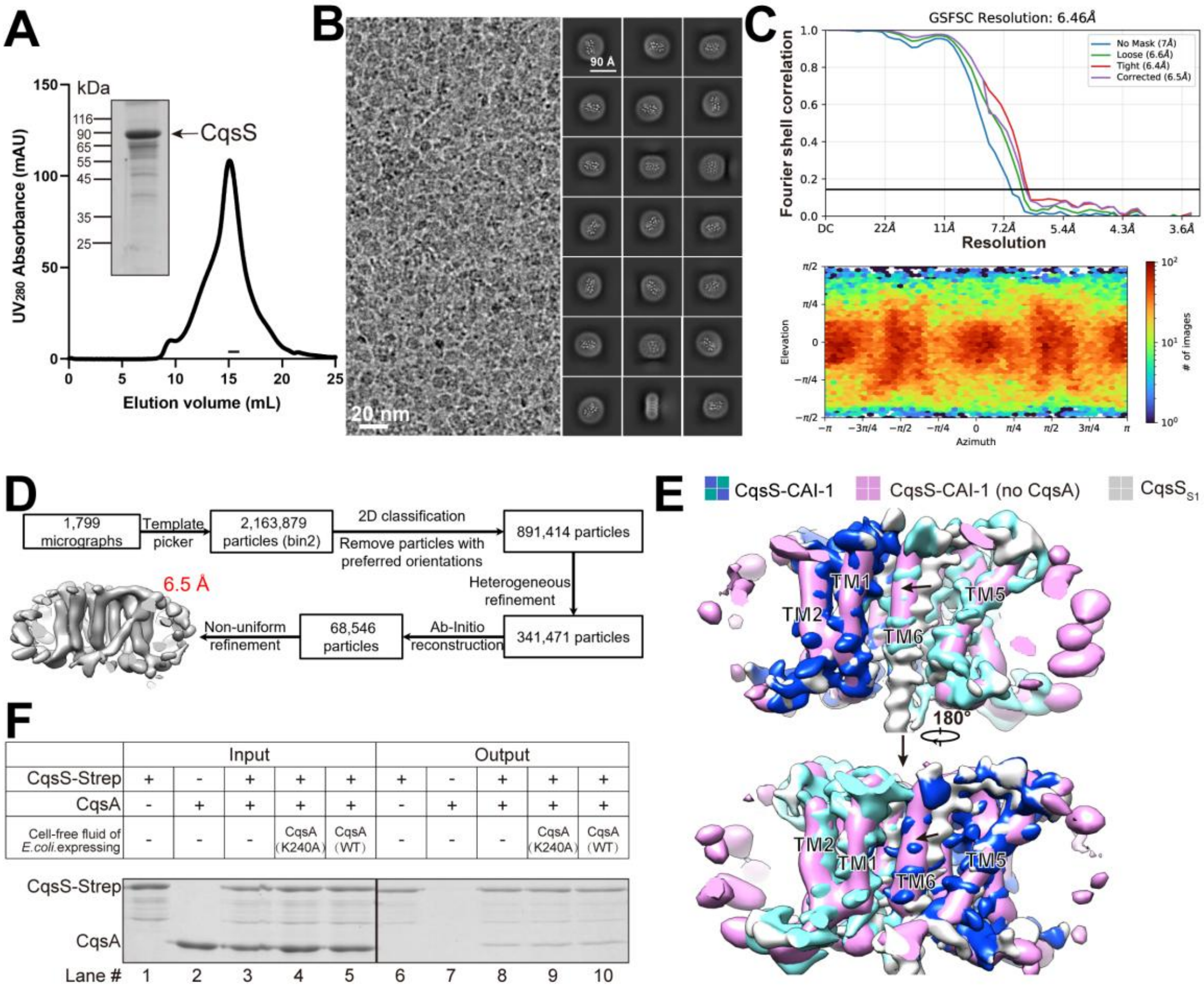


**Fig. S13. Structure determination of the CqsS-CAI-1 complex in the absence of CqsA.** (*A*) Representative SEC profile of the purified CqsS-CAI-1 complex in the absence of CqsA. To prepare this sample, CqsS was coexpressed with the CqsA-F298A/K302A mutant protein, which makes CAI-1 but lacks the ability to interact with CqsS. The purified CqsS protein was thus expected to bind CAI-1 but not CqsA, designated CqsS-CAI-1 (no CqsA). Peak fractions were concentrated for cryo-EM sample preparation. *Inset*: SDS-PAGE analysis of the concentrated protein used for cryo-EM sample preparation. (*B*) Representative micrograph (*left*) and 2D averages

(*right*). (*C*) GSFSC curves (*top*) and angular distribution map (*bottom*) for the final reconstruction. (*D*) Flowchart for the structural determination. (*E*) Comparison of the structures of CqsS-CAI-1, CqsS-CAI-1 (no CqsA), and $CqsS_{S1}$. (*F*) Assessment of the influence of CAI-1 on the interaction between CqsS and CqsA via *in vitro* pull-down assay. Purified CqsS protein was incubated with purified WT CqsA protein, supplemented with Luria-Bertani (LB) medium (lanes 3 and 8) or 10% (v/v) cell-free culture fluid from *E. coli* expressing WT *cqsA* (lanes 5 and 10) or the catalytically-inactive *cqsA* K240A variant (lanes 4 and 9), followed by affinity purification using the Strep tag on CqsS. The input (lanes 1-5) and output (lanes 6-10) of the pull-down assay were subjected to SDS-PAGE analysis.

**Table S1. Summary of data collection and model statistics.**

| | CqsS$_{S1}$ EMD-66349 PDB 9WXL | CqsS$_{S2}$ EMD-66354 PDB 9WXQ | CqsS-ATPγS$_{S1}$ EMD-66361 PDB 9WY6 | CqsS-ATPγS$_{S2}$ EMD-66365 PDB 9WY7 | CqsS-CqsA EMD-66360 PDB 9WY4 | CqsS-CAI-1 EMD-66357 PDB 9WXU |
|---|---|---|---|---|---|---|
| **Data collection and processing** | | | | | | |
| EM | Titan Krios (Thermo Fisher) | | | | | |
| Voltage (kV) | 300 | | | | | |
| Detector | BioQutum K3 (Gatan) | | | | | |
| Electron dose ($e^-/Å^2$) | 50 | | | | | |
| Pixel size (Å) | 0.82 | | | | | |
| Micrographs | 1,630 | | 1,434 | | 2,731 | |
| **Reconstruction** | | | | | | |
| Software | cryoSPARC | | | | | |
| Particles | 124,184 | 137,177 | 150,052 | 140,065 | 130,780 | 131,035 |
| Symmetry | C1 | | | | | |
| Resolution (Å) | 3.2 | 3.3 | 3.5 | 3.5 | 2.9 | 3.4 |
| Map sharpening B factor ($Å^2$) | 120 | 126 | 136 | 139 | 75 | 162 |
| **Model building, Refinement and Validation** | | | | | | |
| Software | Coot, Phenix | | | | | |
| Model composition | | | | | | |
| Protein residues | 1,149 | 1,030 | 1,057 | 1,045 | 2,500 | 336 |
| Nonhydrogen atoms | 8,711 | 7,932 | 8,207 | 8,046 | 19,091 | 2,904 |
| Ligands | 0 | 0 | 1 | 1 | 4 | 2 |
| R.m.s. deviations | | | | | | |
| Bond lengths (Å) | 0.005 | 0.011 | 0.005 | 0.004 | 0.005 | 0.005 |
| Bond angles (°) | 0.84 | 1.12 | 0.79 | 0.80 | 0.76 | 0.95 |
| MolProbity score | 1.80 | 1.81 | 1.91 | 2.21 | 2.28 | 1.99 |
| Clashscore | 7.69 | 9.96 | 8.59 | 11.44 | 9.60 | 10.67 |
| Poor rotamers (%) | 0.60 | 0.64 | 1.11 | 1.78 | 3.63 | 0.67 |
| Ramachandran plot | | | | | | |
| Favored (%) | 94.35 | 95.87 | 93.89 | 93.13 | 95.01 | 93.03 |
| Allowed (%) | 5.38 | 3.94 | 5.83 | 6.39 | 4.71 | 6.97 |
| Disallowed (%) | 0.26 | 0.20 | 0.29 | 0.48 | 0.28 | 0 |

**Movie S1. Conformational changes in CqsS from CqsS-ATPγS$_{S1}$, through CqsS$_{S2}$, CqsS-ATPγS$_{S2}$, to CqsS$_{S1}$.**

The video was generated with the CqsS$_{S1}$, CqsS$_{S2}$, CqsS-ATPγS$_{S1}$, and CqsS-ATPγS$_{S2}$ structures.

**Movie S2. Conformational changes in the TMD between the CqsS apo- and CAI-bound states.**

The video was generated with the structures of CqsS$_{S1}$ and CqsS-CAI-1 as the first and final frames, respectively.

**References**


1 Zheng, S. Q. *et al.* MotionCor2: anisotropic correction of beam-induced motion for improved cryo-electron microscopy. *Nat Methods* **14**, 331-332, doi:10.1038/nmeth.4193 (2017).

2 Grant, T. & Grigorieff, N. Measuring the optimal exposure for single particle cryo-EM using a 2.6 A reconstruction of rotavirus VP6. *Elife* **4**, e06980, doi:10.7554/eLife.06980 (2015).

3 Tunyasuvunakool, K. *et al.* Highly accurate protein structure prediction for the human proteome. *Nature* **596**, 590-596, doi:10.1038/s41586-021-03828-1 (2021).

4 Emsley, P., Lohkamp, B., Scott, W. G. & Cowtan, K. Features and development of Coot. *Acta Crystallogr D Biol Crystallogr* **66**, 486-501, doi:10.1107/s0907444910007493 (2010).

5 Adams, P. D. *et al.* PHENIX: a comprehensive Python-based system for macromolecular structure solution. *Acta Crystallogr D Biol Crystallogr* **66**, 213-221, doi:10.1107/S0907444909052925 (2010).

6 Schrodinger, LLC. *The PyMOL Molecular Graphics System, Version 1.8* (2015).

7 Pettersen, E. F. *et al.* UCSF Chimera--a visualization system for exploratory research and analysis. *J Comput Chem* **25**, 1605-1612, doi:10.1002/jcc.20084 (2004).

8 Higgins, D. A. *et al.* The major Vibrio cholerae autoinducer and its role in virulence factor production. *Nature* **450**, 883-886, doi:10.1038/nature06284 (2007).
